# Motivational interviewing for alcohol use reduction in Thai patients

**DOI:** 10.1515/med-2025-1307

**Published:** 2025-10-27

**Authors:** Rattikorn Muangnang, Mullika Singhasuriya, Amâncio António de Sousa Carvalho

**Affiliations:** Psychiatric and Mental Health Nursing, Boromarajonani College of Nursing, Udon Thani, Thailand; Psychiatric and Mental Health Nursing, Udon Thani Hospital, Udon Thani, Thailand; Health School, University of Trás-os-Montes and Alto Douro, Quinta de Prados, 5000-801, Vila Real, Portugal

**Keywords:** alcohol drinking, alcoholism, motivation, patients, outpatient clinics

## Abstract

**Background and aim:**

Alcohol consumption is a major public health issue, linked to a wide range of physical, psychological, familial, and social harms, as well as increased rates of violence, accidents, and mortality. The aim of this study is to evaluate the effectiveness of a motivational interviewing program in reducing alcohol consumption patterns among Thai patients.

**Methods:**

This quasi-experimental study included a control group (CG) and an experimental group (EG), each consisting of 30 patients. Data were collected using a structured questionnaire and analyzed with SPSS, utilizing both descriptive and inferential statistical methods.

**Results:**

The majority of patients in the CG were aged 45–60 years, whereas the majority in the EG were aged 25–44 years. The intervention involved 4 sessions over 8 weeks motivational interviewing program (MIP). The mean AUDIT scores in the CG were 24.10 before the intervention and 22.90 after, while in the EG, scores decreased from 22.63 to 19.33. The interaction between the factors (CG and EG) before the intervention did not have a significant effect (Multivariate Analysis of Variance, MANOVA: *p* = 0.255), but was significant after the intervention (MANOVA: *p* = 0.009).

**Conclusion:**

MIP had a significant effect on reducing alcohol consumption among patients of the EG.

## 1 Introduction

The consumption of alcoholic beverages presents a significant public health concern due to the extensive physical, psychological, familial, and social harm it inflicts on individuals, their families, and society at large. The severity of this issue is reflected in its extensive and lasting repercussions [1].

Global per capita alcohol consumption (APC) declined slightly from 5.7 L in 2010 to 5.5 L in 2019, reflecting a 4.5% reduction. In 2019, the highest APC was recorded in the World Health Organization (WHO) European Region (9.2 L), followed by the Americas (7.5 L). That year, 17% of individuals aged 15 and older and 38% of current drinkers engaged in binge drinking – consuming at least 60 g of pure alcohol on one or more occasions in the past month – while continuous heavy drinking remained prevalent among men (6.7%). Total APC trends vary across WHO regions, with a significant decline in Europe and a notable increase in South-East Asia since 2000 [2], where Thailand is located.

According to the National Health Examination Survey, the prevalence of alcohol consumption in Thailand declined from 72.6% in 2003/2004 to 55.9% in 2014 among males and from 35.1 to 23.0% among females. However, in 2019/2020, a slight increase was observed, with prevalence rates rising to 59.0% among males and 31.0% among females [3].

Alcohol remains the most widely consumed psychoactive substance with considerable public health implications. The widespread acceptance of alcohol use, combined with social norms that encourage drinking behavior, often leads to the minimization of the adverse effects of alcohol on the population’s health. These detrimental consequences are frequently overshadowed by arguments emphasizing the purported benefits of alcohol consumption for well-being and economic development. The presence of conflicting narratives regarding the harms and benefits of alcohol production and consumption may hinder timely health-seeking behaviors and undermine initiatives aimed at mitigating the associated health and social burdens [2].

Alcohol consumption ranks among the top ten risk factors contributing to disease and disability worldwide [4]. The WHO’s global strategy reflects a broad international consensus, recognizing the reduction in harmful alcohol use and its associated health and social burdens as a critical public health priority. Within this strategic framework, the concept of harmful alcohol use is comprehensive, encompassing drinking behaviors that lead to adverse health and social consequences for the individual, those in their immediate environment, and society at large. Additionally, it includes patterns of alcohol consumption that are linked to an increased risk of negative health outcomes [5].

In 2019, alcohol consumption was responsible for approximately 2.6 million deaths globally, accounting for 4.7% of all fatalities that year. The burden of alcohol-attributable disease is disproportionately higher among males, with 2 million alcohol-related deaths and 6.9% of all disability-adjusted life years (DALYs) attributed to alcohol use. In contrast, among females, alcohol consumption contributed to 0.6 million deaths and accounted for 2.0% of all DALYs in the same period.

The WHO African and European regions exhibit the highest rates of alcohol-attributable deaths per 100,000 individuals. Although global alcohol consumption and its associated harms have declined to some extent since 2010, the health and social burden resulting from alcohol use remains significantly high. Younger populations are disproportionately affected, with individuals aged 20–39 years accounting for the highest proportion of alcohol-attributable deaths, representing 13% of all such fatalities in 2019 [2].

Globally, an estimated 400 million individuals, or 7% of the world’s population aged 15 years and older, are affected by alcohol use disorders, while approximately 209 million people (3.7% of the global adult population) experience alcohol dependence. The prevalence of alcohol use disorders varies considerably across different WHO regions. Since 2010, a declining trend in the global prevalence of alcohol use disorders has been observed, primarily driven by reductions in the Americas, Europe, and the Western Pacific. Conversely, an increasing trend has been reported in the African, Eastern Mediterranean, and South-East Asia regions.

The age-standardized burden of mortality (death rates) and morbidity (DALY rates) associated with alcohol consumption per liter consumed is highest in low-income countries, followed by lower-middle-income countries, and lowest in high-income countries [2].

Harmful alcohol consumption accounts for 5.3% of all global deaths and 5.0% of all DALYs. The social burden associated with harmful substance use is considerable, often linked to adverse health outcomes, educational impairment, and an increased propensity for crime and unemployment. This burden disproportionately affects underdeveloped countries, where inadequate infrastructure limits the capacity to address the consequences of substance use effectively. According to the World Mental Health Survey, between 76 and 85% of individuals with severe mental illnesses (including substance use disorders, anxiety, or mood) in low-and middle-income countries (LMICs) did not receive treatment for their conditions over a 12 month period [6].

Alcohol consumption is a significant contributor to chronic disease and injury and ranks among the top five risk factors for mortality and disability worldwide. Its adverse effects are responsible for approximately 3.3 million deaths and 139 million DALYs lost annually on a global scale. Additionally, alcohol use is closely linked to high-risk behaviors, including criminal activity, aggressive driving, interpersonal violence, and self-inflicted injury, all of which pose substantial harm not only to individuals but also to society at large. Compared to high-income countries, LMICs report higher prevalence rates of hazardous drinking patterns, such as binge drinking and episodic heavy drinking, as well as an earlier initiation of alcohol consumption [7].

In 2021, the economic cost of alcohol consumption in Thailand was estimated at 165,450.5 million baht, representing 1.02% of the country’s Gross Domestic Product and amounting to 2,500 baht per capita. Indirect costs constituted the majority of this financial burden (96.32%), with an estimated value of 159,358.8 million baht, whereas direct costs were assessed at 6,091.7 million baht. Among indirect costs, premature mortality emerged as the most significant component, accounting for 95.45% of the total economic impact, with an estimated cost of 157,918.7 million baht (134,424.7 million baht for males and 23,493.9 million baht for females). In 2021, alcohol-attributable premature deaths were estimated at 22,804 cases (19,678 males and 3,127 females), resulting in 666,393 years of life lost. Direct costs, which amounted to 6,091.7 million baht (3.7% of the total cost), were primarily driven by healthcare expenditures estimated at 4,370.1 million baht (2.7% of the total cost). Additionally, the justice system incurred costs of 1,704.3 million baht (1.03% of the total cost), while property damage-related expenses were estimated at 17.2 million baht (0.01% of the total cost) [3].

Motivational interviewing (MI) is a widely recognized brief treatment strategy and recovery technique initially developed to support individuals experiencing alcohol use disorders. It is a direct, client-centered approach aimed at enhancing intrinsic motivation for behavioral change by addressing and resolving ambivalence. MI employs a conversational style designed to facilitate behavioral modifications that promote improved health outcomes. Grounded in the principles of motivational psychology, this therapeutic method fosters internally driven, rapid changes. Treatment within the MI framework is characterized by a non-authoritarian, empathetic approach that encourages individuals to take personal responsibility for their own transformation. It incorporates objective and personalized assessment results to evaluate the severity of problematic behaviors, provides explicit guidance on recommended courses of action, and offers a structured set of options for change. Additionally, MI utilizes motivational strategies to activate clients’ internal resources, thereby strengthening their commitment to sustained behavioral improvement [5,8,9].

According to other authors [10], MI operates through the interaction of four fundamental processes: engaging, focusing, evoking, and planning. These processes are conceptualized as overlapping, sequential, and recursive. Engaging refers to the establishment of a supportive and collaborative relationship between the practitioner and the client, fostering a conducive environment for meaningful interaction. Focusing entails directing the discussion toward a specific agenda, ensuring that the conversation aligns with the individual’s concerns and goals. The third process, evoking, involves eliciting the client’s intrinsic motivation for change, encouraging them to articulate their own reasons and arguments for behavioral transformation. Finally, planning encompasses the development of a concrete commitment to change, coupled with the formulation of a structured action plan to facilitate the implementation of desired behavioral adjustments.

MI is widely employed in the treatment of substance use and abuse. Its widespread adoption may be attributed to the brevity and perceived cost-effectiveness of MI strategies. As a highly targeted approach, MI facilitates behavioral change and guides individuals through the stages of transformation [11]. It is designed to support individuals at varying levels of readiness for change, including those who may initially exhibit resistance to modification [12].

Several studies on this topic have been conducted in Thailand. However, as highlighted by the WHO, while policy interventions serve as essential and cost-effective measures to mitigate alcohol-related harm, the implementation of contextually appropriate and effective patient-level interventions is equally necessary for the development of comprehensive harm reduction strategies [7]. This study was designed to address this need by contributing to the development of comprehensive strategies for reducing alcohol-related harm.

It is within this context that the present study was developed, with the objective of evaluating the effectiveness of a MI program (MIP) in reducing alcohol consumption patterns among Thai patients.

## 2 Methods

This quasi-experimental, longitudinal study utilized a quantitative approach. The research took place at a hospital in Northeastern Thailand, where participants received care in the psychiatric outpatient clinic [13].

The inclusion criteria comprised (i) male patients aged 18–60 years and (ii) individuals receiving care at the outpatient psychiatric clinic. Exclusion criteria included (i) inability to read, write, or communicate effectively in Thai; (ii) alcohol withdrawal symptoms that could interfere with treatment, determined by an Alcohol Withdrawal Scale score below 5; (iii) psychiatric symptoms such as hallucinations, paranoia, delusions, or significant difficulty in regulating thoughts, emotions, and behaviors; and (iv) severe comorbid conditions preventing study participation, including alcohol-related physical illnesses requiring hospitalization.

The study population consisted of patients diagnosed with Alcohol Dependence (ICD-10 code: F10.2) by psychiatrists, based on the criteria established by the WHO. This diagnosis corresponds to Alcohol Use Disorder according to the criteria outlined in the Diagnostic and Statistical Manual of Mental Disorders, Fifth Edition, Text Revision (DSM-5-TR) [14]. In 2023, approximately 491 patients met these diagnostic criteria.

The sample was selected using a non-random approach. A convenience sampling method was used to assign 30 patients to the Experimental Group (EG), who participated in a MIP. Another 30 patients were placed in the Control Group (CG), receiving standard care based on hospital protocols. For ethical considerations, the MIP program was extended to the CG after the study concluded.

The data collection instrument comprised three components: (i) sociodemographic information, including age, marital status, educational level, and occupational status; (ii) the Alcohol Use Disorders Identification Test (AUDIT) scale, designed to assess alcohol consumption patterns; and (iii) the SOCRATES-8A Scale, which measured motivation to quit drinking. In this study, only the AUDIT Scale was utilized.

The AUDIT is a 10-item screening tool developed by WHO to evaluate alcohol consumption, drinking behaviors, and alcohol-related problems. It was adapted by the Department of Mental Health, Ministry of Public Health, and translated into Thai by Silpakit and Kittirattanapaiboon [15]. The AUDIT consists of ten questions covering three key domains: hazardous alcohol use (three questions), consisting of frequency of drinking, typical quantity, and frequency of heavy drinking; harmful alcohol use (four questions), encompassing guilt after drinking, blackouts, alcohol-related injuries, and others concerned about drinking; and alcohol dependence (three questions), which includes features such as impaired control over drinking, increased salience of drinking, and morning drink. Risk Level 1 (Low-risk consumption): 0–7 points; Risk Level 2 (Risky consumption): 8–15 points; Risk Level 3 (Harmful consumption): 16–19 points; and Risk Level 4 (Probable alcohol dependence): 20 points or higher. The tool demonstrated strong reliability, evidenced by a Cronbach’s alpha coefficient of 0.86.

Data were collected by the researchers through questionnaire administration during patients’ psychiatric outpatient consultations in a suitable setting. The data collection period spanned from December 2023 to March 2024. Patients were informed of the study’s objectives and invited to participate voluntarily. They were also assured that they could withdraw at any time without facing any negative consequences. Informed consent was obtained before participation.

The MIP was supported for the reduction in alcohol consumption among individuals with alcohol dependence. This program consisted of four sessions over 8 weeks, with each session lasting approximately 15–30 min and including four activities, as described below.

Activity 1 (Week 1): Assessment, motivation-building, and personalized feedback. Patients underwent an evaluation of their drinking behaviors and their motivation to quit alcohol consumption. They received individualized feedback based on their assessment results, including an analysis of the severity of their alcohol-related issues.

Activity 2 (Weeks 2–3): Strengthening commitment to change. Patients explored their confidence in quitting alcohol, declared their commitment, and discussed the pros and cons of drinking vs abstaining. Misconceptions were addressed, and additional information was provided. The session concluded with a collaborative summary of the change plan.

Activity 3 (Weeks 4–7): Setting goals and taking steps for self-change. Patients reviewed their goals and change plan, addressed obstacles, and explored key motivational factors. The researcher reinforced their commitment, boosted confidence in quitting alcohol, and provided hope and encouragement throughout the process.

Activity 4 (Week 8): Setting goals and sustaining self-change (follow through). Patients reviewed their goals and change plan, revisited motivational factors, and reinforced their commitment to quitting alcohol. The researcher provided guidance on behavior management, boosted confidence, and encouraged long-term adherence to the change. At the conclusion of the final session, the questionnaire was re-administered for the second round of data collection.

One of the researchers, with experience in implementing MI programs, accompanied the patients in the four activities of this MI program, ensuring that the MIP was adequately implemented in the EG.

The database was processed using the Statistical Package for the Social Sciences (SPSS, version 25.0). Data analysis included both descriptive and inferential statistics. For descriptive analysis, absolute and relative frequencies, mode calculations for all variables, and measures of central tendency and dispersion for scalar variables were examined. Regarding inferential analysis, the formulated hypothesis was tested using the Multivariate Analysis of Variance (MANOVA) to compare the two moments of AUDIT scale application (before and after the MIP) across both study groups. The effectiveness of the MIP in reducing alcohol consumption was further assessed using mixed repeated-measures Analysis of Variance (ANOVA). Statistically significant differences were considered at a probability level of *p* < 0.05 [16].

**Ethical approval:** The ethical principles outlined in the Declaration of Helsinki and Vancouver were upheld, including privacy, anonymity, confidentiality, and conflict of interest. A favorable opinion was granted by the Ethics Committee of the Hospital (UDH REC No.108/2566) on December 4, 2023.

## 3 Results

The largest group of patients in the CG (*n* = 30) was in the age range of 45–60 years (56.7%), while in the EG (*n* = 30), the largest group was in the 25–44 age range (53.3%). The largest age group of 45–60 years represented 51.7% of the total sample (*n* = 60). Regarding marital status, the majority of patients had a partner, with 76.7% in the CG, 86.7% in the EG, and 81.7% in the total sample, showing a significant proportion in the EG. In terms of educational level, most patients had completed primary education, with 60.0% in the CG, 70.0% in the EG, and 65.0% in the total sample. Finally, concerning occupational status, the majority of patients were employed, with 53.3% in the CG, 50.0% in the EG, and 51.7% in the total sample, with a slightly higher percentage in the CG (Table 1).

**Table 1:** Sociodemographic characterization of the sample by group type

Variables	CG		EG		Total	
	Af	Rf (%)	Af	Rf (%)	Af	Rf (%)
**Age group**						
25–44 years old	13	43.3	16	53.3	29	48.3
45–60 years old	17	56.7	14	46.7	31	51.7
**Marital status**						
Without a partner	7	23.3	4	13.3	11	18.3
With a partner	23	76.7	26	86.7	49	81.7
**Educational level**						
Primary school	18	60.0	21	70.0	39	65.0
Secondary school	7	23.3	5	16.7	12	20.0
Bachelor degree or higher	5	16.7	4	13.3	9	15.0
**Occupation**						
Unemployed	14	46.7	15	50.0	29	48.3
Employed	16	53.3	15	50.0	31	51.7

Legend: Af – Absolute frequency; CG – Control group; EG – Experimental group; Rf – Relative frequency.

To assess whether the distribution of sociodemographic variables differed significantly between the two groups under analysis (CG and EG), a chi-square test was conducted to evaluate the association between group type and, successively, age group, marital status, educational level, and occupation. The results indicate that no statistically significant differences were observed between group type and age group (*χ*², *p* = 0.606), marital status (*χ*², *p* = 0.506), educational level (*χ*², *p* = 0.461), or occupation (*χ*², *p* = 1.000).

The majority of patients in both the CG and the EG, as well as the overall sample, initiated alcohol consumption between the ages of 16 and 20, accounting for 63.3% of participants. The mean age at which patients in the EG began consuming alcohol was 19.27 ± 2.59 years. Regarding alcohol consumption frequency, the majority of patients in the CG and the total sample reported consuming alcohol more than four times per week, with proportions of 73.3 and 51.7%, respectively. In contrast, the majority of patients in the EG reported consuming alcohol two to three times per week (53.3%), indicating a lower frequency compared to the other groups. In terms of daily alcohol consumption, most patients in the CG consumed five or more standard drinks (83.4%), as did the total sample (63.3%). Conversely, in the EG, the majority of patients consumed three to four standard drinks per day (56.7%). Regarding beverage preferences, white spirits were the most commonly consumed alcoholic beverages across all groups, reported by 83.3% of patients in the CG, 66.7% in the EG, and 75.0% in the total sample, with a higher prevalence in the CG (Table 2).

**Table 2:** Characterization of alcohol consumption behavior by group type and total sample

Variables	CG		EG		Total	
	Af	Rf (%)	Af	Rf (%)	Af	Rf (%)
**Age of beginning drinking**						
Lower than 15 years old	4	13.3	8	26.7	12	20.0
16–20 years old	19	63.3	19	63.3	38	63.3
21 years old and over	7	23.4	3	10.0	10	16.7
**Alcohol consumption frequency**						
2–4 times a month	0	0.0	5	16.7	5	8.3
2–3 times a week	8	26.7	16	53.3	24	40.0
4 or more times a week	22	73.3	9	30.0	31	51.7
**Daily consumption quantity**						
1–2 standard drinks	1	3.3	0	0.0	1	1.7
3–4 standard drinks	4	13.3	17	56.7	21	35.0
5 or more standard drinks	25	83.4	13	43.3	38	63.3
**Type of alcoholic beverage**						
White spirits	25	83.3	20	66.7	45	75.0
Colored liquor and others	5	16.7	10	33.3	15	25.0

Legend: Af – Absolute frequency; CG – Control group; EG – Experimental group; Rf – Relative frequency.

Regarding the AUDIT scale score categories, in the CG prior to the program, the majority of patients were classified under the probable dependence category (83.4%), with no patients falling into the free consumption category (0.0%). Following the program, the majority remained in the probable dependence category (53.3%), though the proportion in this category declined significantly. Notably, no patients were classified within the free consumption category post-intervention (0.0%).

Conversely, in the experimental group (EG), prior to the program, the majority of patients were classified under the probable dependence category (56.7%), with no individuals falling within the free consumption category (0.0%). Following the intervention, the majority of patients transitioned to the harmful consumption category (83.4%), indicating a significant shift from probable dependence to harmful consumption. Notably, no patients remained in the free consumption category post-program (0.0%) (Table 3).

**Table 3:** Distribution of AUDIT categories by group and total sample (%)

Variables	CG		EG		Total	
	Af Rf (%)		Af Rf (%)		Af Rf (%)	
**Pre-program**						
Free consumption	0	0.0	0	0.0	0	0.0
Risky consumption	1	3.3	0	0.0	1	16.7
Harmful consumption	4	13.3	13	43.3	17	28.3
Probable dependence	25	83.4	17	56.7	42	70.0
**Post-program**						
Free consumption	0	0.0	0	0.0	0	0.0
Risky consumption	2	6.7	1	3.3	3	5.0
Harmful consumption	12	40.0	25	83.4	37	61.7
Probable dependence	16	53.3	4	13.3	20	33.3

Legend: Af – Absolute frequency; CG – Control group; EG – Experimental group; Rf – Relative frequency.

An analysis of the mean AUDIT score revealed a decline in both the CG and the EG from the pre-program to the post-program phase. However, this reduction was more pronounced in the EG, with a decrease of 3.30 points compared to 1.20 points in the CG (Table 4).

**Table 4:** Descriptive statistics of the CG and EG before and after program implementation

AUDIT scores	CG		EG	
	Af	Mean value ± SD	Af	Mean value ± SD
Pre-program	30	24.10 ± 5.061	30	22.63 ± 4.817
Post-program	30	22.90 ± 6.036	30	19.33 ± 3.942

Legend: Af – Absolute frequency; CG – Control group; EG – Experimental group; SD – Standard deviation.

The MANOVA test assessing the mean AUDIT score before and after the implementation of the MIP, with group type (CG and EG) as a fixed factor, revealed a statistically significant effect of group type on the composite AUDIT score across both time points (Pillai’s Trace = 0.183; *F*(2,57) = 6.399; *p* = 0.003; Power = 0.887). This finding aligns with the results obtained from the repeated measures ANOVA test (Table 5).

**Table 5:** Results of the MANOVA multivariate tests

Effect	Value	*F*	df	Error	Sig.	OP
Intercept Pillai’s trace	0.959	659.812	2.000	57.000	0.000	1.000
Group Pillai’s trace	0.183	6.399	2.000	57.000	0.003	0.887

Legend: F – Snedecor’s F Distribution; OP – Observed power; Sig. – Significance level.

The test of effects between subjects revealed that the group type factor did not have a statistically significant effect on the AUDIT score before the implementation of the MIP (*p* = 0.255), but had a very significant effect after the implementation of the MIP (MANOVA: *p* = 0.009), with the mean value of the EG being the one that decreased the most, that is, the risk consumption decreased in the patients in this group. In CG also, it decreased after the implementation of the MIP, but this decrease was less pronounced. This sharper decrease may have been due to the implementation of the MIP.

## 4 Discussion

In the present study, prior to the implementation of the MIP, the majority of patients in both the CG and EG were categorized as having probable dependence, with the proportion in the CG exceeding that in the EG by 30 percentage points. Following the MIP, the percentage of patients classified under probable dependence declined in both groups; however, the reduction was more pronounced in the EG, where it decreased by 43 percentage points, compared to a 30-point reduction in the CG.

These findings are not consistent with those reported in a study conducted in Paris, France [17], which involved a sample of 85 individuals diagnosed with Alcohol Use Disorder. In that study, participants were divided into two groups: one receiving MI (*n* = 37) and the other receiving standard treatment (*n* = 48). Prior to the intervention, the percentage of patients classified in the probable dependence category was lower in the CG (60.1%) compared to the EG (83.3%), indicating a difference of approximately 23 percentage points. In contrast, the present study observed a higher percentage of probable dependence in the CG than in the EG, thereby reversing the pattern observed in the Paris study. This discrepancy suggests that the two studies involved patient samples with differing baseline characteristics and patterns of alcohol consumption. Specifically, in the comparison study [17], patients in the CG exhibited lower levels of alcohol dependence at the outset.

A study conducted in Rio de Janeiro, Brazil [18], involving a sample of 30 alcoholic patients, employed a quasi-experimental single-group design to assess changes before and after the intervention program. Findings from that study indicated that the probable dependence category was entirely eliminated following the implementation of the MIP. In contrast, in the present study, the proportion of patients in this category decreased significantly, reaching 13%. While both studies demonstrated a reduction in probable dependence, the decline observed in the comparative study appears to have been more pronounced.

In the study conducted in Paris, France [17], the pre-program AUDIT score was 21.27 ± 9.11 points for the CG and 26.08 ± 6.36 points for the EG. These values were lower than those observed in the present study for the CG and significantly lower for the EG. Consequently, in the Paris study, the proportion of patients in the EG exhibiting a dependent consumption pattern exceeded that in the CG, whereas in the present study, this pattern was reversed. Notably, post-program AUDIT scores were not reported in the Paris study.

The results of the multivariate analyses (MANOVA and repeated measures ANOVA) conducted in the present study indicate a more substantial and statistically significant reduction in AUDIT scores within the EG, suggesting that the MIP was effective in reducing risky alcohol consumption patterns. Similar findings were reported in studies conducted in Thailand [19,20], where the authors concluded that both counseling programs and motivational enhancement programs contributed to a reduction in risky alcohol consumption. Studies conducted in other countries [17,18] likewise demonstrated that interventions such as brief intervention and the MIP led to a significant decrease in alcohol consumption.

Additionally, a study carried out in Córdoba, Spain [21], involving 268 patients with risky alcohol consumption, found that the MIP was more effective in promoting faster and more sustained behavioral changes compared to conventional health advice, leading to a greater reduction in alcohol consumption within the EG. Furthermore, the previously cited meta-analysis [7] highlighted that brief intervention and MI techniques were the most frequently evaluated approaches and consistently demonstrated positive effects on alcohol-related outcomes. Another meta-analysis examining various interventions for individuals with substance use disorders reviewed a study that compared MI with no intervention or non-MI-based controls over a 3 month follow-up period in samples of adult drinkers. The findings indicated reductions in alcohol use and significant mean effects on abstinence in favor of MI [22].

Based on the results of the multivariate analyses, it can be concluded that the MIP seems to have been effective in reducing alcohol consumption patterns among patients in the sample and served as a successful intervention in achieving the study objective.

The primary limitation of this study pertains to the use of a convenience sample rather than a randomized selection, which may have affected the generalizability of the findings to the broader population of alcohol-dependent individuals receiving treatment in an outpatient clinic.

This study may have significant implications for the professional practice of the healthcare team, particularly the nursing staff at the outpatient clinic, within the context of this research. The demonstrated effectiveness of the MIP has motivated its adoption in the treatment of these patients. Given that MI is a non-pharmacological intervention that is easy to implement and free of adverse effects, its integration into clinical practice could yield tangible benefits for patients at this clinic. Notably, the healthcare team has already implemented the MIP within the CG, thereby extending the positive effects of this intervention to a group that had not received it during the study.
